# A Bionic Knee Exoskeleton Design with Variable Stiffness via Rope-Based Artificial Muscle Actuation

**DOI:** 10.3390/biomimetics10070424

**Published:** 2025-07-01

**Authors:** Shikai Jin, Bin Liu, Zhuo Wang

**Affiliations:** School of Mechanical Engineering, Northwestern Polytechnical University, Xi’an 710072, China; shikaijin@mail.nwpu.edu.cn (S.J.); lbin@mail.nwpu.edu.cn (B.L.)

**Keywords:** knee exoskeleton, artificial muscle, elastic actuator, variable stiffness mechanism

## Abstract

This paper presents a novel design for a bionic knee exoskeleton equipped with a variable stiffness actuator based on rope-driven artificial muscles. To meet the varying stiffness requirements of the knee joint across different gait modes, the actuator dynamically switches between multiple rope bundle configurations, thereby enabling effective stiffness modulation. A mathematical model of the knee exoskeleton is developed, and the mechanical properties of the selected flexible aramid fiber ropes under tensile loading are analyzed through both theoretical and experimental approaches. Furthermore, a control framework for the exoskeleton system is proposed. Wearable experiments are conducted to evaluate the effectiveness of the variable stiffness actuation in improving compliance and comfort across various gait patterns. Electromyography (EMG) results further demonstrate that the exoskeleton provides a compensatory effect on the rectus femoris muscle.

## 1. Introduction

Robotic exoskeletons with powered actuators can augment or restore human movement [1]. In recent years, due to the impaired walking ability from aging, injury, or neurological disorders, lower-limb exoskeletons have garnered significant attention as a means to assist human mobility in various contexts [2,3,4]. In the healthcare sector, such devices are expected to improve rehabilitation outcomes and reduce long-term care costs for patients with neurological injuries or age-related mobility decline [5,6]. They are also being explored in industrial and military domains to enhance human performance—for example, enabling workers to handle heavy loads or perform repetitive tasks with reduced risk of musculoskeletal injury [7,8,9,10]. The knee joint, being the largest and most complex joint in the human body, generates stiffness that meets the needs of human movement through the interaction of skeletal muscles, connective tissue, and other soft tissues. This stiffness not only bears the weight of the body but also needs to meet the needs of human movement. Knee weakness or injury can lead to instability and degenerative changes in the joint, underscoring the necessity of effective knee exoskeletons for rehabilitation and mobility support [11]. Therefore, there is a strong and growing need for advanced knee exoskeleton designs that can aid movement for the elderly and disabled, facilitate gait rehabilitation after injuries, and augment healthy users in physically demanding jobs.

Researchers have developed a variety of bionic exoskeletons to meet the biomechanical needs of the knee joint, and a range of actuation strategies have emerged [12,13,14]. Early exoskeleton designs often employed rigid actuation, using high-torque electric motors or linear hydraulic drive coupled with gear transmissions to directly drive the knee joint [15,16,17]. This approach delivers the required joint torque but results in a stiff actuation with no compliance. To improve safety and adaptability, many designs incorporate compliant elements in their actuators, such as springs, soft materials, magnetic and pneumatic components, etc. By introducing elastic elements into the drive system and timely adjusting the stiffness of the drive structure, exoskeletons can absorb shock and provide a measure of compliance, making the interaction with the user’s leg more comfortable than a purely rigid drive. These designs are also more in line with the laws of human biomechanics.

Despite the progress in actuation technology for wearable devices, existing knee exoskeletons still face notable limitations. The major challenge is the lack of adaptability across different gait phases and activities. Many current devices operate with a fixed stiffness or pre-defined assistance profile that cannot seamlessly accommodate different mechanical requirements of various gaits [18,19]. A stiff actuator tuned for weight support in running gait may impede the natural leg motion during swing in walking gaits, whereas a freely moving joint during walking swing may not provide adequate support for running or uphill climbing at heel strike. While some designs introduce traditional elastic components to achieve some compliance, their stiffness is typically fixed by design and not easily changeable in real time, limiting adaptability. Moreover, adding multiple springs or conventional variable stiffness mechanisms can introduce mechanical complexity and huge mass, making the exoskeleton heavier and potentially less reliable. Among VSAs, representative examples [20,21,22] include the MACCEPA (Mechanically Adjustable Compliance and Controllable Equilibrium Position Actuator), pneumatic actuators, and other SEAs. While these approaches have demonstrated efficacy in modulating joint stiffness, they often suffer from certain limitations when applied to wearable knee exoskeletons—such as added weight, bulky mechanical components, and complex control schemes. For instance, MACCEPA typically requires external linkages and levers, which increase the mechanical complexity and make integration into compact knee joint structures challenging. Pneumatic systems, though capable of providing tunable compliance, require external air supply units, making them less suitable for fully portable or lightweight wearable designs. So far, the actuation strategies of most existing exoskeletons do not adequately mimic the behavior of human muscle.

Biologically inspired design provides a promising approach to addressing these challenges. Unlike traditional actuators, human muscles are capable of modulating joint stiffness and exhibit viscoelastic behavior, allowing them to rapidly transition between compliant and rigid states. In the knee joint, the quadriceps femoris group plays a key role in both generating extension torque and maintaining joint stability by adjusting its activation level. This natural ability to vary stiffness and respond adaptively to different motion demands serves as the conceptual basis for the artificial muscle actuation strategy proposed in this study. When a low force or stiffness is required, only a small subset of muscle fibers is activated. As greater force or stiffness is needed, additional motor units from smaller to larger are sequentially recruited. The human neuromuscular system modulates joint stiffness and impedance by varying how many muscle fibers are engaged and their activation levels, rather than relying on a single, always-stiff element [23]. This elegant natural mechanism provides a template for engineering an actuator that could overcome the limitations noted above.

In this paper, we present a bionic knee exoskeleton design based on rope-driven artificial muscles. The proposed exoskeleton is actuated by a novel variable stiffness system that dynamically recruits different combinations of parallel elastic elements, analogous to how the quadriceps recruits additional muscle fibers. Specifically, the actuator consists of a bundle of rope-like tensile elements arranged in parallel. By engaging or disengaging individual ropes via a controllable clutch mechanism, the effective stiffness and output torque of the actuator can be tuned in discrete steps. For example, during low-load conditions such as walking gait, only one rope or thinner ropes might be engaged, making the joint compliant and minimizing resistance. During high-load conditions such as walking uphill or climbing stairs, more ropes can be recruited, dramatically increasing the joint stiffness and force output available at the knee joint. This variable recruitment actuation strategy endows the exoskeleton with adaptable gait dynamics: the joint’s mechanical characteristics can change in real time to match the biomechanical demands of different gait phases or activities. Additionally, the ability to modulate stiffness can enhance safety and performance: the exoskeleton can be compliant when interacting with the user or terrain to absorb shocks, and stiff when needed for stability. This adaptability is expected to improve the exoskeleton’s gait compatibility, making assisted walking feel more natural. By closely mimicking the adaptive stiffness behavior of human muscles, the proposed design seeks to bridge the gap between rigid robotic assistance and the fluid, context-dependent support provided by human muscles.

The remainder of this paper is organized as follows. Section 2 outlines the biomechanical principles underlying variable stiffness in human muscles and introduces the rope-based artificial muscle actuator design of the bionic knee exoskeleton. Section 3 details the mechanical design and theoretical modeling of the proposed bionic knee exoskeleton. Section 4 presents the mechanical properties analysis of flexible ropes. Section 5 presents the mechatronics and control strategy of the knee exoskeleton system. Section 6 introduces the prototype of the knee exoskeleton and conducts a series of wearable experiments to evaluate the effectiveness of the design. Finally, Section 7 concludes the paper with a summary of contributions.

## 2. Rope-Based Artificial Muscle Actuator Design

**Figure 1:**
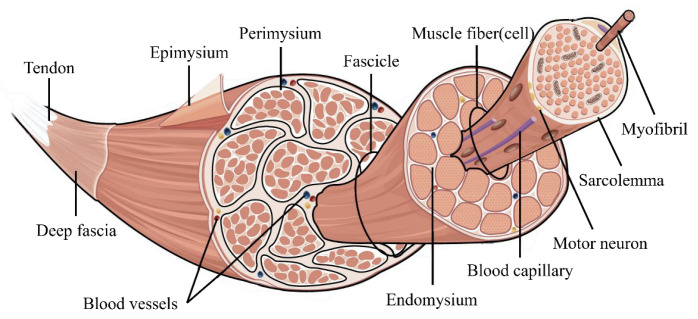
Schematic diagram of the human muscle physiological structure.

Human muscle movement is driven by the contraction of individual muscle fibers. As illustrated in Figure 1, reproduced from [24], muscles consist of bundles of muscle fibers, with each fiber containing myofibrils. In human physiology, joint stiffness is dynamically adjusted through the selective activation of muscle fibers by the nervous system. This neuromuscular regulation enables muscles to modulate force output and mechanical compliance during movement. Specifically, increased recruitment of muscle fibers surrounding a joint leads to higher joint stiffness, while reduced activation leads to decreased stiffness. This intricate mechanism enables the body to effectively modulate joint stability and flexibility during movement.

**Figure 2:**
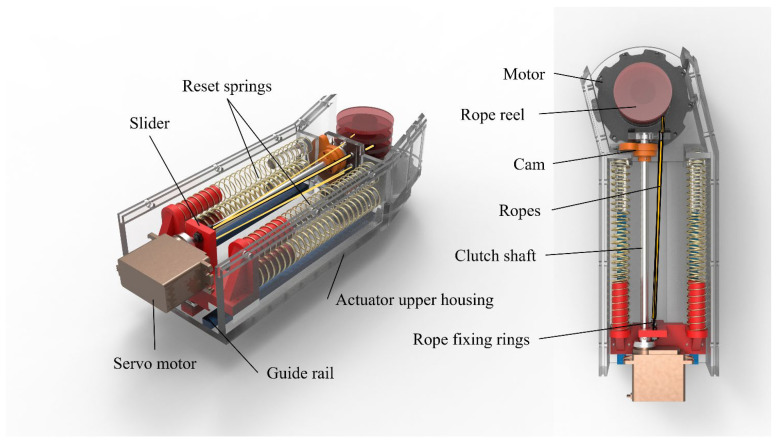
Schematic diagram of the actuator.

Inspired by the mechanism, we designed an actuator that uses fiber ropes to simulate muscle fibers and imitates the effect of muscle recruitment by pulling a combination of rope bundles of different sizes. The schematic diagram of the actuator is shown in Figure 2, which consists of a 3D print housing, a motor, four reset springs, a slider, two sets of guide rails, a rope reel, a clutch including a servo motor, a cam, a clutch shaft, and ropes, etc. When the motor is running, its rotation is transferred to the rope reel, which separates each rope at a certain distance and drags all the ropes. The ropes undergo elastic–plastic deformation and transmit power, thereby providing linear displacement. When the slider is driven in a linear direction by the ropes, it compresses the four integrated reset springs nested inside the slider. When the motor reverses direction and the rope bundle relaxes, the elastic restoring force of the springs drives the slider back to its initial position, effectively completing the retraction process.

**Figure 3:**
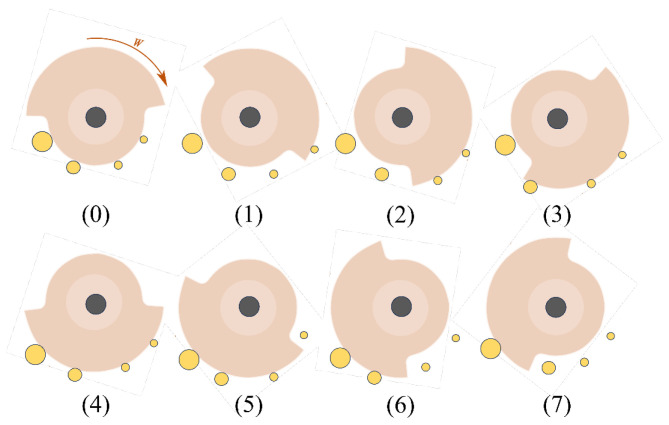
Schematic diagram of the clutch working principle.

The clutch realizes the bionic muscle unit by controlling the number of ropes connected, thereby achieving rapid switching of the overall stiffness. More specifically, the rotation of the cam driven by the servo motor enables sequential engagement of multiple locking positions, thereby completing the locking action of the fiber ropes tied to the slider. Theoretically, this type of clutch enables switching between multiple rope bundle configurations. Owing to its fan-shaped layout, the number of achievable configurations increases proportionally with the number of ropes. Considering the stiffness resolution required by the knee exoskeleton and the need to balance performance with structural complexity, this study adopts a clutch configuration with four ropes, enabling seven combinations. While increasing the number of ropes would allow for finer stiffness modulation, it would also increase the size and complexity of the clutch mechanism, making it less suitable for integration into compact wearable systems. Given that the target motion scenarios—including walking, running, stair ascent, and ramp ascent—do not demand extremely high stiffness resolution, the current configuration was selected as an optimal compromise between functional sufficiency and mechanical simplicity. Through the schematic diagram of the clutch switching principle in Figure 3, the clutch activation process of multiple groups of rope bundles can be seen more clearly.

As shown in Figure 3, four ropes connected to the rope reel are sized 0.5 mm, 0.5 mm, 1 mm, and 2 mm, respectively. The clutch shaft rotates the cam to sequentially limit the locking terminal and realize the sequential access of four ropes. By continuing to rotate, each rope can be unlocked in reverse order. When the servo motor drives the cam to rotate nearly one circle, seven rope bundle combinations can be switched. The specific combinations are listed in Table 1.

The clutch engages the rope bundles via a cam mechanism driven by a servo motor. The selected servo can rotate over a 180° range in approximately 2.5 ms. Standard control of the servo requires a base control pulse of about 20 ms. Taking into account the signal transmission delay of the main control board and actuation processing time, the overall response time of the system falls within the same order of magnitude as the natural recruitment time of muscle fibers. Through the designed drive clutch mechanism, different total stiffnesses can be achieved based on multiple sets of flexible rope bundle sequences to adapt to different movement patterns in daily life.

## 3. Bionic Knee Exoskeleton Structural Design and Modeling

**Figure 4:**
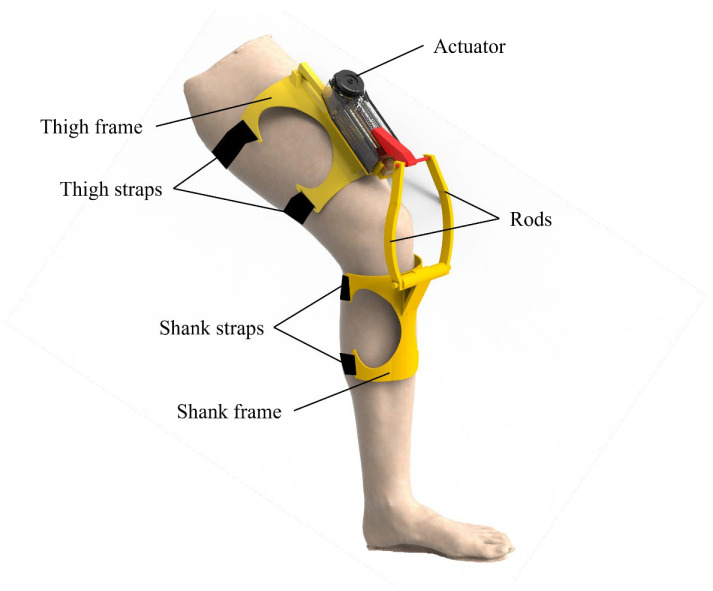
Schematic diagram of the knee exoskeleton.

Based on the concept of adaptive stiffness inspired by human muscle behavior, we have designed a bionic knee exoskeleton using a rope-driven artificial muscle actuator. The schematic diagram of the knee exoskeleton is demonstrated in Figure 4. The exoskeleton is mainly composed of an actuator, thigh frame, shank frame, and rods. When the actuator applies linear motion, the rods transmit power to the shank frame, providing joint torque for the knee exoskeleton.

**Figure 5:**
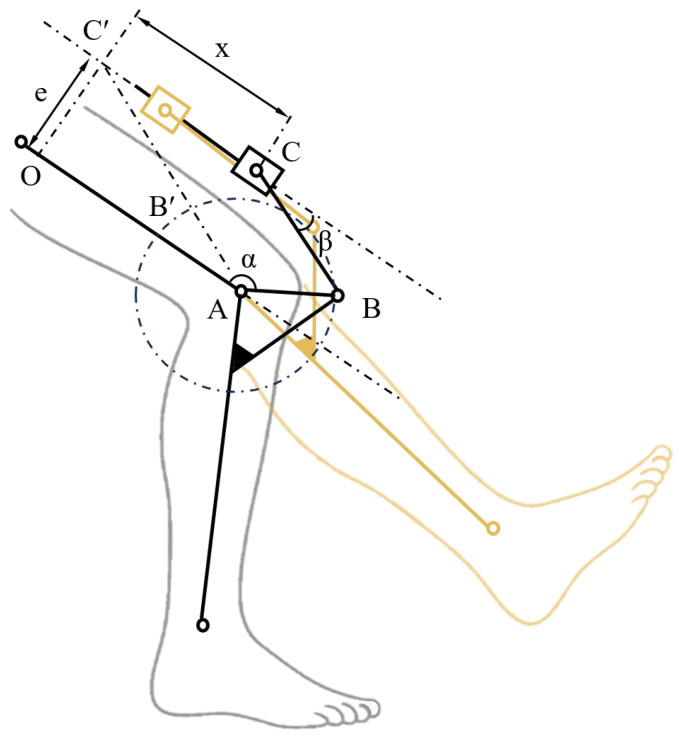
Mechanism diagram of the knee exoskeleton.

According to the mechanism diagram of the knee exoskeleton in Figure 5, the mathematical model of the exoskeleton is established. Since the deformation of each component of the knee exoskeleton device is minimal under the load scenario, each component is considered an absolute rigid body for the purpose of modeling.

(1) $$l_{AO}=\sqrt{{\left(l_{AB^{\prime }}+l_{B^{\prime }C^{\prime }}\right)}^{2}-e^{2}}=\sqrt{{\left(l_{AB}+l_{BC}\right)}^{2}-e^{2}},$$

where e represents the distance from O to C’, $l_{AO}$ represents the distance from A to O, and the others are defined similarly. As shown in Figure 5, let angle α be the rotation angle of point B on the crank AB, and let angle β be the angle between the connecting rod BC and the motion trajectory of the slider C.

For any given angle α and β

(2) $$x=\sqrt{{\left(l_{AB}+l_{BC}\right)}^{2}-e^{2}}-(l_{AB}\cos\alpha +l_{BC}\cos\beta ),$$

(3) $$e=l_{BC}\sin\beta +l_{AB}\sin\alpha ,$$

(4) $$\sin\beta =\frac{e-l_{AB}\sin\alpha }{l_{\mathrm{BC}}},$$

To facilitate the derivation, we define ratios $m=\frac{e}{l_{\mathrm{BC}}}$ and $n=\frac{l_{BC}}{l_{AB}}$, thus $\sin\beta =m-\frac{\sin\alpha }{n}$.

(5) $$\mathrm{As}\;\sin^{2}\beta +\cos^{2}\beta =1,\cos\beta =\sqrt{1-{\left(m-\frac{\sin\alpha }{n}\right)}^{2}}$$

Then, substituting the expression of cosβ into Equation (1):

(6) $$x=\sqrt{{\left(l_{\mathrm{AB}}+l_{\mathrm{BC}}\right)}^{2}-e^{2}}-(l_{\mathrm{AB}}\cos\alpha +l_{\mathrm{BC}}\sqrt{1-{\left(m-\frac{\sin\alpha }{n}\right)}^{2}}),$$

The displacement *x* can be expressed as

(7) $$x=\sqrt{{\left(l_{\mathrm{AB}}+l_{\mathrm{BC}}\right)}^{2}-e^{2}}-l_{\mathrm{AB}}\left[\cos\alpha +n\sqrt{1-{\left(m-\frac{\sin\alpha }{n}\right)}^{2}}\right],$$

To obtain the velocity equation of the slider, the displacement *x* is differentiated:

$$\frac{dx}{dt}=\frac{dx}{d\alpha }\times \frac{d\alpha }{dt},\;\mathrm{the}\;\mathrm{angle}\;\mathrm{velocity}\;\omega =\frac{d\alpha }{dt}.$$

Thus, the velocity can be expressed as

(8) $$v(t)=\frac{dx}{dt}=\omega l_{\mathrm{AB}}\left[\sin\alpha -\frac{\left(m-\frac{\sin\alpha }{n}\right)\cos\alpha }{\sqrt{1-{\left(m-\frac{\sin\alpha }{n}\right)}^{2}}}\right],$$

Since the linear movement of point C is pulled by the flexible rope, the deformation of the rope is determined by its elastic deformation and plastic deformation. We can regard the flexible rope as a spring with a variable elastic coefficient in practical applications. The relationship between the tension on the rope and its elongation is shown in the equation below.

(9) Ftension=MklΔlt+Δt,

where *F*_tension_ represents the magnitude of the tension on the flexible rope at time *t*; *M* is the maximum breaking force of the rope, treated as a constant during tension; *k* represents the dynamic stiffness; *l* represents the instantaneous length of the rope at time t; Δt is a very short time interval; and $\Delta l_{t+\Delta t}$ represents the total deformation of the rope after the instantaneous time increment. Therefore, the instantaneous length of the rope at time t+Δt is

(10) $$l_{t+\Delta t}=l+\Delta l_{t+\Delta t},$$

Because each instantaneous time period Δt is taken as 1‰ of the complete gait cycle in actual calculation, the deformation increment within an instantaneous time increment can be ignored, so Equation (9) can be simplified as follows:

(11) $$F_{\mathrm{tension}}=\frac{Mk}{l+\Delta l}\Delta l,$$

The variable elastic coefficient of the stretched fiber rope can be approximately expressed as follows:

(12) $$K=\frac{Mk}{l+\Delta l},$$

When multiple ropes are connected, the total variable elastic coefficient *K* of the system is equal to the sum of the elastic coefficients of each rope:

(13) $$K=K_{1}+K_{2}+\ldots +K_{n},$$

Thus, the passive force generated by the rope during driving is as follows:

(14) $$F_{\mathrm{passive}}=K\cdot l_{\mathrm{total}},$$

where $l_{\mathrm{total}}$ represents the total deformation length of the rope.

Considering that in the drive system, the motor is directly connected to the rope reel, and all ropes are pulled by the rotation of the rope reel, the angular velocity of the motor can be obtained as follows:

(15) ωm=v(t)rr,

where $\omega_{m}$ represents the motor angular velocity and $r_{r}$ represents the radius of the rope reel.

Thus, the torque required by the motor can be expressed as follows:

(16) $$P_{m}=\frac{F_{m}v(t)}{\eta }=\frac{F_{m}r_{r}\omega_{m}}{\eta }=\frac{T_{m}\omega_{m}}{\eta },$$

(17) $$T_{m}=\frac{P_{m}}{\omega_{m}}\eta ,$$

where $P_{m}$ represents the power output of the motor, $F_{m}$ represents the traction force of the motor, and η represents the overall transmission efficiency from the motor’s power input to the mechanical output at the actuator’s terminal. This includes energy loss from rope reel winding and unwinding, negative work by the reset springs, and all kinds of friction losses in the drive system.

## 4. Mechanical Properties Analysis of the Ropes

Since the mechanical properties of the flexible ropes directly influence the actuator’s ability to emulate the adaptive stiffness behavior of human muscles—and thereby affect the driving performance of the knee exoskeleton—the analysis of the mechanical response characteristics of the flexible rope is particularly important.

### 4.1. Rope Selection

**Figure 6:**
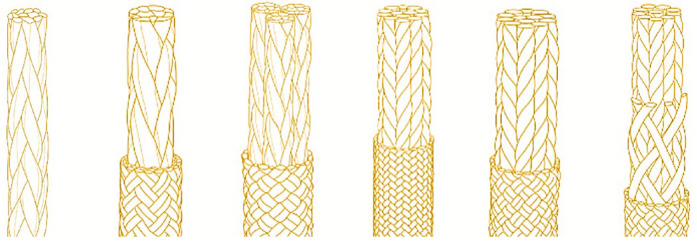
Schematic diagram of various aramid fiber ropes.

The bundle structure of the fiber rope is very similar to the structure of muscle fiber groups. After comparing the properties of many types of fiber ropes, we chose aramid fiber for the actuator in this study. Aramid fiber ropes, also known as Kevlar fiber ropes [25], have lower density and higher strength than steel wires, and also have the characteristics of dimensional stability, fatigue resistance, and corrosion resistance. The aramid fiber rope structure, as shown in Figure 6, provides sufficient strength and stability while having the elasticity and durability required to simulate the movement of muscle fibers.

These properties give aramid fiber ropes great potential as artificial muscle materials, which are expected to improve the performance and practicality of artificial muscles.

### 4.2. Stress–Strain Modeling of Aramid Rope

**Figure 7:**
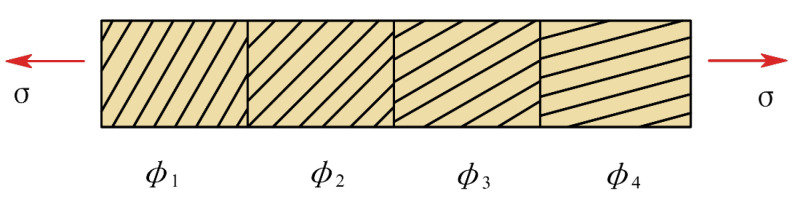
Schematic diagram of the microscopic molecular chain of aramid fiber.

Northolt et al. [26] used the onset of a sequential and plastic orientation mechanism of the chains brought about by the resolved shear stress to explain the yield phenomenon of composite fibers on the tensile curve. Under tensile conditions, the fiber is considered to be composed of parallel rows of identical fibrils that are uniformly stressed along the fiber axis. Each fibril consists of a series of rectangular domains connected end to end, as shown in Figure 7.

The molecular orientation of aramid fibers plays a decisive role in their stress-deformation process. The macroscopic deformation of aramid fibers is a concentrated reflection of the microscopic molecular elongation and molecular chain torsion, that is, $\varepsilon_{\mathrm{total}}=\varepsilon_{\mathrm{tension}}+\varepsilon_{\mathrm{torsion}}$. The $\varepsilon_{\mathrm{tension}}+\varepsilon_{\mathrm{reversible}}$ part participates in the nonlinear viscoelastic behavior as the stretching time and load change, while the $\varepsilon_{\mathrm{tension}}+\varepsilon_{\mathrm{irreversible}}$ part represents the viscoplastic behavior of the fiber material. Since the viscoplastic strain accumulates only when the flow criterion *F* ≥ *F*_max_ is satisfied, it depends on the loading time, the current stress, and the maximum historical tensile stress [27].

Comprehensively considering the behavior under tension, the strain of aramid fiber rope is mainly manifested in the following aspects:

(18) $$\varepsilon (t,\sigma )=\left\{\begin{array}{c} \begin{array}{l} \varepsilon_{tension}(\sigma )\\ + \end{array}\\ \varepsilon_{torsion}(t,\sigma )\left\{\begin{array}{c} \varepsilon_{reversible}(t,\sigma )\\ +\\ \varepsilon_{irreversible}(t,\sigma ) \end{array}\right. \end{array}\right.$$

It is assumed that the total strain ε(t) generated during the tensile process of aramid fiber can be divided into reversible nonlinear viscoelastic strain $\varepsilon_{ve}(t)$ and an irreversible viscoplastic strain $\varepsilon_{vp}(t)$, that is:

(19) $$\varepsilon (t)=\varepsilon_{ve}(t)+\varepsilon_{vp}(t),$$

In order to systematically establish the stress–strain relationship of aramid rope under tension, this paper uses the Schapry numerical calculation theory [28] for the reversible viscoelastic strain and uses Perzyna’s one-dimensional model for viscoplastic strain to describe the irreversible viscoplastic strain [29,30]. Schapery’s model offers a generalized nonlinear viscoelastic framework that can accurately describe the creep and recovery behavior observed in high-performance polymers and composite fibers under finite strain. Perzyna’s model is well-suited to capture the rate-dependent yielding and viscoplastic flow observed in aramid fibers under high-load or high-strain-rate conditions. According to Schapry’s viscoelastic theory, the integral equation of the viscoelastic strain of aramid fiber rope at constant temperature is

(20) $$\varepsilon_{ve}^{t}=g_{0}^{\sigma^{t}}D_{{\,}_{0}}\sigma^{t}+g_{1}^{\sigma^{t}}\int_{0}^{t}\Delta D(\Psi^{t}-\Psi^{\tau })\frac{d(g_{2}^{\sigma^{t}}\sigma )}{d\tau }d\tau ,$$

The effective time *ψ* can be defined as follows:

(21) $$\Psi^{t}\equiv \Psi (t)=\int_{0}^{t}\frac{d\xi }{a_{\sigma }^{\sigma^{\xi }}a_{T}^{T}},$$

where $g_{0}$, $g_{1}$, $g_{2}$, and $a_{\sigma }$ are all nonlinear stress functions of the material, and their magnitudes are only related to the stress on the fiber rope. Under low stress levels, $g_{1}=g_{2}=a_{\sigma }=1$, at which time the deformation–recovery characteristics of the aramid rope are completely dependent on its viscoelastic characteristics. In this study, in order to simplify the model, we ignore the effect of temperature on the mechanical properties of the fiber rope and set $a_{T}^{T}$ to 1. △*D* is the uniaxial transient flexibility of the material, and its Prony series is expressed as follows:

(22) $$\Delta D^{\Psi^{t}}=\sum_{n=1}^{N}D_{n}(1-e^{-\lambda_{n}\Psi^{t}}),$$

where N is the number of terms in the Prony series, $D_{n}$ is the coefficient of the nth term, and $\lambda_{n}$ is the reciprocal of the nth hysteresis time. Putting Equation (22) into Equation (20), we obtain

(23) $$\varepsilon_{ve}^{t}=g_{0}^{t}D_{0}\sigma^{t}+g_{1}^{t}g_{2}^{t}\sum_{n=1}^{N}D_{n}-g_{1}^{t}\sum_{n=1}^{N}D_{n}q_{n}^{t},$$

(24) $$\sqrt{q_{n}^{t}=\int_{0}^{t-\Delta t}e^{\left[-\lambda_{n}(\Psi^{t}-\Psi^{\tau })\right]}\frac{d(g_{2}^{\tau }\sigma^{\tau })}{d\tau }d\tau +\int_{t-\Delta t}^{t}e^{\left[-\lambda_{n}(\Psi^{t}-\Psi^{\tau })\right]}\frac{d(g_{2}^{\tau }\sigma^{\tau })}{d\tau }d\tau },$$

The following definitions are obtained:

(25) $$\Delta \Psi^{t}\equiv \Psi^{t}-\Psi^{t-\Delta t},$$

Then Equation (24) can be decomposed as

(26) $$\int_{0}^{t-\Delta t}e^{\left[-\lambda_{n}(\Psi^{t}-\Psi^{\tau })\right]}\frac{d(g_{2}^{\tau }\sigma^{\tau })}{d\tau }d\tau =e^{-\lambda_{n}\Delta \Psi^{t}}q_{n}^{t-\Delta t},$$

(27) $$\int_{t-\Delta t}^{t}e^{\left[-\lambda_{n}(\Psi^{t}-\Psi^{\tau })\right]}\frac{d(g_{2}^{\tau }\sigma^{\tau })}{d\tau }d\tau =\frac{1-e^{-\lambda_{n}\Delta \Psi^{t}}}{\lambda_{n}\Delta \Psi^{t}}(g_{2}^{t}\sigma^{t}-g_{2}^{t-\Delta t}\sigma^{t-\Delta t}),$$

Combining Equations (27) and (24), we get

(28) $$q_{n}^{t}=e^{-\lambda_{n}\Delta \Psi^{t}}q_{n}^{t-\Delta t}+\frac{1-e^{-\lambda_{n}\Delta \Psi^{t}}}{\lambda_{n}\Delta \Psi^{t}}(g_{2}^{t}\sigma^{t}-g_{2}^{t-\Delta t}\sigma^{t-\Delta t}),$$

The total strain can be obtained by putting Equation (28) into Equation (23) as follows:

(29) $$\begin{array}{c} \varepsilon_{ve}^{t}=\left[g_{0}^{t}D_{0}\sigma^{t}+g_{1}^{t}g_{2}^{t}\sum_{n=1}^{N}D_{n}-g_{1}^{t}g_{2}^{t}\sum_{n=1}^{N}D_{n}\frac{1-e^{-\lambda_{n}\Delta \Psi^{t}}}{\lambda_{n}\Delta \Psi^{t}}\right]\sigma^{t}\\ \;\;-g_{1}^{t}\sum_{n=1}^{N}D_{n}\left[e^{-\lambda_{n}\Delta \Psi^{t}}q_{n}^{t-\Delta t}-g_{2}^{t-\Delta t}(1-e^{-\lambda_{n}\Delta \Psi^{t}})\frac{\sigma^{t-\Delta t}}{\lambda_{n}\Delta \Psi^{t}}\right]\\ \;\;\equiv {\tilde{D}}^{t}\sigma^{t}-f^{t} \end{array},$$

(30) $$f^{t}=g_{1}^{t}\sum_{n=1}^{N}D_{n}\left[q_{n}^{t}-g_{2}^{t}\sum_{n=1}^{N}D_{n}\frac{1-e^{-\lambda_{n}\Delta \Psi^{t}}}{\lambda_{n}\Delta \Psi^{t}}\sigma^{t}\right],$$

Then, the strain increment is obtained as follows:

(31) $$\begin{array}{c} \Delta \varepsilon^{t}=\varepsilon^{t}-\varepsilon^{t-\Delta t}\\ \;\;=\left({\tilde{D}}^{t}\sigma^{t}-{\tilde{D}}^{t-\Delta t}\sigma^{t-\Delta t}\right)-(f^{t}-f^{t-\Delta t})\\ \;\;={\tilde{D}}^{t}\sigma^{t}-{\tilde{D}}^{t-\Delta t}\sigma^{t-\Delta t}\\ \;\;-\sum_{n=1}^{N}D_{n}\left[\begin{array}{l} (g_{1}^{t}e^{-\lambda_{n}\Delta \Psi^{t}}-g_{1}^{t-\Delta t})q_{n}^{t-\Delta t}\\ -g_{2}^{t-\Delta t}\sigma^{t-\Delta t}(g_{1}^{t-\Delta t}\frac{1-e^{-\lambda_{n}\Delta \Psi^{t-\Delta t}}}{\lambda_{n}\Delta \Psi^{t-\Delta t}}-g_{1}^{t}\frac{1-e^{-\lambda_{n}\Delta \Psi^{t}}}{\lambda_{n}\Delta \Psi^{t}}) \end{array}\right] \end{array},$$

As proposed by Perzyna, the one-dimensional model of the viscoplastic strain of aramid fibers can be a function of stress and time. The total plastic strain $\varepsilon_{vp}^{t}$ is the accumulation of all plastic strain increments $\Delta \varepsilon_{vp}^{t}$, that is

(32) $$\varepsilon_{vp}^{t}=\sum_{i=1}^{n}\Delta \varepsilon_{vp}^{t},$$

(33) $$\Delta \varepsilon_{vp}^{t}=\varepsilon_{vp}^{t+\Delta t}-\varepsilon_{vp}^{t},$$

(34) $$\varepsilon_{vp}^{t}=\sigma_{t}D_{p}(\sigma )t^{m(\sigma )},$$

where $D_{p}$ and m are the plastic strain factors of the material. By taking the derivative of Equation (34), the instantaneous increment of plastic strain $\Delta \varepsilon_{vp}^{t}$ can be expressed as follows:

(35) $$\Delta \varepsilon_{vp}^{t}=\sigma_{t}D_{p}(\sigma )mt^{m(\sigma )-1}\Delta t,$$

Then, under the maximum plastic stress, the plastic strain of the fiber rope can be quantified as follows:

(36) $$\varepsilon_{vp}^{t}=\sum_{i=1}^{n}\sigma_{t}D_{p}(\sigma )mt^{m(\sigma )-1}\Delta t=\int_{0}^{t}\sigma_{t}D_{p}(\sigma )m\tau^{m(\sigma )-1}d\tau ,$$

### 4.3. Experimental-Based Rope Model Parameter Determination

In combination with the above theoretical model of viscoelasticity and viscoplasticity of aramid fibers, it is necessary to determine the relevant parameters in the model [31]. The undetermined parameters in the model are summarized in Table 2 below.

**Figure 8:**
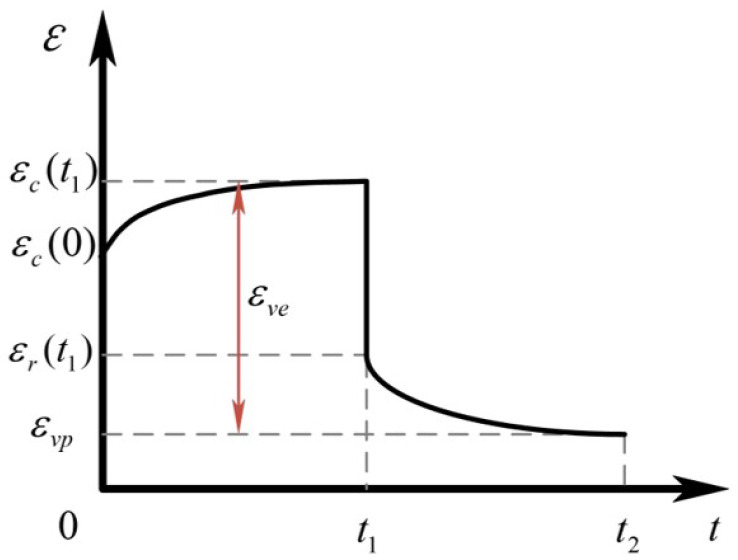
Deformation–recovery curve of the aramid rope.

The deformation–recovery curve of the aramid rope is shown in Figure 8.

Generally, the elastic stress–strain relationship of aramid fiber rope can be expressed as follows:

(37) $$\varepsilon_{vp}=g_{2}^{\sigma^{t}}\sigma^{t}\sum_{n=1}^{N}D_{n}\left[e^{-\lambda_{n}(t-t_{1})}-e^{-\lambda_{n}(\frac{t_{1}}{a^{\sigma }}+t-t_{1})}\right],$$

(38) $$g_{1}^{\sigma^{t_{1}}}=\frac{\varepsilon_{c}(t_{1})-\varepsilon_{c}(0)-\varepsilon_{vp}}{\varepsilon_{r}(t_{1})-\varepsilon_{vp}},$$

Aramid fiber ropes exhibit strong viscoelastic properties at low stress levels. In this case, the material parameters $g_{0}$, $g_{1}$, $g_{2}$, and $a_{\sigma }$ are approximately equal to 1. Based on the deformation–recovery data obtained by the experiment, the coefficients $D_{n}$, $\lambda_{n}$, and $g_{1}$ can be determined by fitting the relevant parameters of Equation (37) and Equation (38), respectively.

Based on the research of Lai and Bakker [32], $g_{0}$ and $D_{0}$ of the viscoelastic model can be obtained by fitting Equation (39) under a higher stress level during the experiment.

(39) $$\begin{array}{l} \varepsilon_{cr}=\varepsilon_{c}(t_{1})-\varepsilon_{r}(t)\\ =g_{0}\sigma \sum_{n=1}^{N}D_{n}\left[1-e^{-\lambda_{n}g_{1}(t_{1}/a_{\sigma })}-e^{-\lambda_{n}(t-t_{1})}+e^{-\lambda_{n}(t_{1}/a_{\sigma }+t-t_{1})}\right] \end{array}$$

Similarly, by fitting Equation (37) at a higher stress level, the parameters $g_{2}$ and $a_{\sigma }$ can be obtained. The total strain during the experiment can be expressed as follows:

(40) $$\varepsilon_{c}(t)=g_{0}D_{0}\sigma +g_{1}g_{2}\sigma \sum_{n=1}^{N}D_{n}(1-e^{-\lambda_{n}(\frac{t_{1}}{a_{\sigma }})})+D_{p}\sigma t^{m},$$

By fitting Equation (40), the viscoplastic parameters $D_{p}$ and m of the aramid fiber rope can be determined. So far, all parameters can be achieved.

### 4.4. Experiment of Aramid Fiber Ropes

Five kinds of aramid fiber ropes with rope diameters of 0.5 mm, 0.8 mm, 1.0 mm, 1.5 mm, 2.0 mm, and 2.5 mm were prepared for the experiment. Their parameters are shown in Table 3.

**Figure 9:**
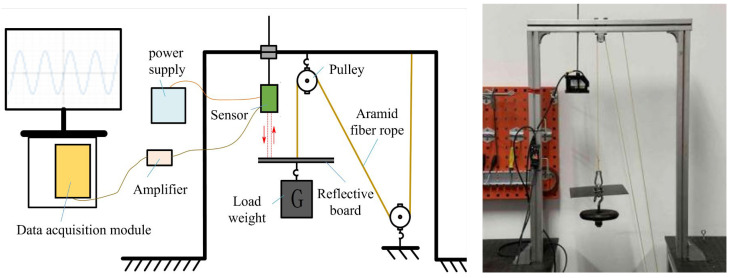
Diagram of the experimental setup.

The experimental setup is shown in Figure 9. The IL300 laser displacement sensor was used in conjunction with the IL1000 sensor amplifier to accurately measure the deformation of the aramid fiber rope at each moment (Keyence, Osaka, Japan). The PCI-1716 data acquisition card and LabView were used for data acquisition and export. After completing the calibration, the experiment was started.

During the cyclic loading experiment of the aramid fiber rope, for different rope diameters, each loading increment is about 2.5% of the rope’s breaking tension, until the loading reaches the level of 20% of the breaking tension. In order to ensure that the fiber rope recovers completely after loading, a 15-min recovery time is given to the fiber rope after loading. Since coefficients $g_{0}$, $g_{1}$, $g_{2}$ and $a_{\sigma }$ have different characteristics under different stress levels, the load stress of each rope diameter needs to be divided into low stress level and high stress level. All coefficients are obtained by fitting the equations in Section 3. To simplify the fitting, let $\lambda_{1}=1$ and construct a geometric sequence with a ratio of 0.1 to assign $\lambda_{1},\lambda_{2},\ldots ,\lambda_{n}$. Taking the rope diameter of 2.5 mm as an example, the fitting results are shown in Table 4. Among them, the stress on the rope is the independent variable *x*, and the corresponding function value is the specific value of each coefficient under this stress. The other five rope diameter coefficients are processed similarly.

**Figure 10:**
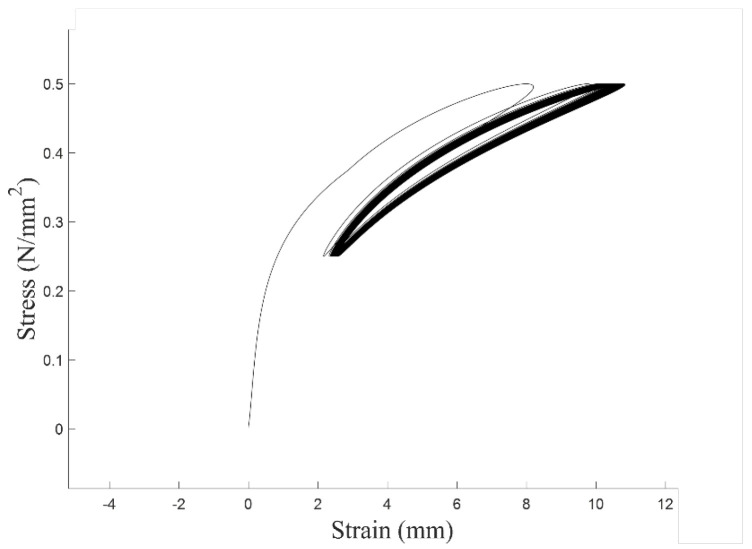
Diagram of the stress–strain cycling curve.

The aramid fiber rope serves as the primary load-bearing element in our actuation system. Its strength and durability are crucial to the stability and safety of the equipment, and also have a profound impact on the application potential of this research in clinical and industrial fields. In order to verify the stability of the mechanical properties of aramid fiber ropes during cyclic loading, a 500-cycle simulation was performed with a single cycle period of 2 s. The stress–strain cycling curve of aramid fiber rope with 2.5 mm diameter is shown in Figure 10.

It is observed that at the initial stage of cyclic loading, the stress–strain loops exhibit relatively wide spacing, indicating that the braided structure of the aramid fiber rope is initially unstable. As the number of cycles increases, the loops become progressively denser, suggesting that the stress–strain slope of the aramid fiber rope gradually approaches a steady-state value. This behavior suggests that the stiffness-softening effect is confined to the early stages of use, and once the aramid rope has undergone this initial pre-conditioning, its stiffness remains consistent in subsequent cycles. Thus, under normal operating conditions, this effect does not lead to a continuous decline in stiffness over time, ensuring the long-term consistency and reliability of actuator performance. In order to quantitatively describe the stress–strain relationship of aramid fiber ropes, we introduce the concept of dynamic stiffness and define it as *k*:

(41) $$k=\frac{(T_{n}^{p}-T_{n-1}^{t})/M}{\varepsilon_{n}^{p}-\varepsilon_{n-1}^{t}},$$

where $T_{n}^{p}$ represents the peak tension of the aramid fiber rope during the nth cyclic loading, $T_{n-1}^{t}$ represents the valley tension during the n−1th cyclic loading, *M* represents the maximum breaking force, $\varepsilon_{n}^{p}$ represents the peak strain value during the nth cyclic loading, and $\varepsilon_{n-1}^{t}$ represents the valley strain value during the (n − 1)th cyclic of loading [33,34]. After a sufficient number of cycles, when the dynamic stiffness approaches a steady-state value, it can be reformulated a

(42) $$k=\frac{T/M}{\varepsilon }=\frac{T/M}{\Delta l/l},$$

(43) $$T=\frac{Mk}{l}\Delta l$$

**Figure 11:**
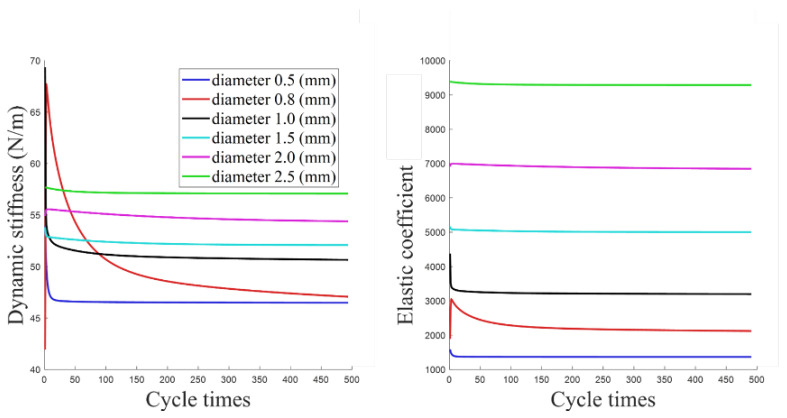
Dynamic stiffness and elastic coefficient diagram.

Transforming Equation (42) into Equation (43), the *Mk*/*l* can be the elastic coefficient. The dynamic stiffness and elastic coefficient results of aramid fiber ropes with six different diameters are shown in Figure 11.

The results show that as the number of cycles increases, the dynamic stiffness of the aramid fiber rope gradually tends to a stable value, and the value gradually increases with the increase of the rope diameter. The 0.8 mm rope exhibited unstable stiffness characteristics in the early and middle stages of the 500-cycle loading test, which may be due to its special braided structure that resulted in delayed mechanical stability. Ropes of other sizes quickly reached a steady state. In addition, the smaller the diameter of the aramid fiber rope, the more drastic the decrease in its dynamic stiffness at the beginning of the cycle.

## 5. Mechatronics and Control

### 5.1. Mechatronics of the Knee Exoskeleton

**Figure 12:**
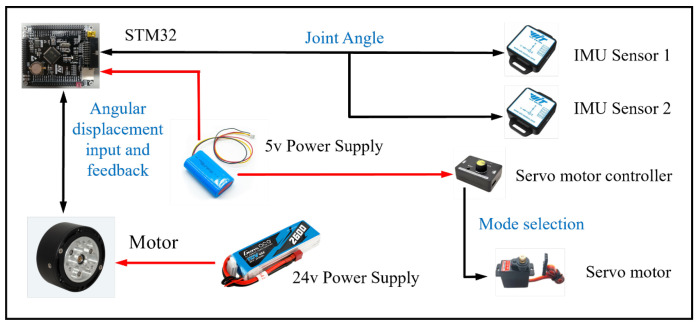
Mechatronics diagram of the knee exoskeleton.

The mechatronics diagram of the knee exoskeleton is shown in Figure 12, where the black lines represent information transmission and the red lines represent power supply. We use stm32 as the control board of the knee exoskeleton to process sensor information and control the motor. Two inertial measurement unit (IMU) sensors are placed flat on the shank frame and thigh frame to realize gait recognition. The drive mode of the actuator is adjusted through the servo motor controller to achieve power output with different stiffness. Walking, running, or stair ascent each triggers a corresponding rope bundle configuration based on predefined settings to achieve stiffness adjustment.

The servo motor drives the cam to change the number of ropes involved in each gait. By calculating the z-axis angle of the two IMU sensors, real-time knee joint angle data can be obtained. According to the gait data collected by the sensor, STM32 calculates the corresponding angular displacement required by the motor and uses the CAN communication protocol to control the motor to achieve trajectory tracking control of the exoskeleton.

### 5.2. Control of the Knee Exoskeleton

Based on the mechatronic setup above, a trajectory-tracking strategy can be implemented on the knee exoskeleton to approach the required displacement of the knee joint. We adopted a basic PID controller to verify the feasibility and effectiveness of the variable stiffness mechanism based on the rope-driven artificial muscle actuator. Gait recognition is mainly achieved by analyzing the feedback from two IMU sensors on the frames of the knee exoskeleton.

**Figure 13:**
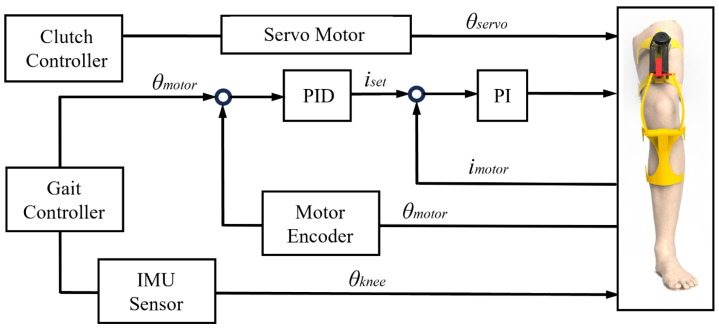
Control schematic of the knee exoskeleton.

A control schematic diagram of the knee exoskeleton is shown in Figure 13. When the exoskeleton is operating, it provides assistance to the knee joint during extension according to the joint angle required for gait. The clutch controller controls the servo to change the *θ*_servo_ to achieve different stiffness requirements under different movement modes or walking speeds. The gait controller controls the motor to the corresponding angular displacement according to the gait information, and makes real-time adjustments based on the feedback from the motor encoder.

## 6. Wearable Experiment of the Knee Exoskeleton

**Figure 14:**
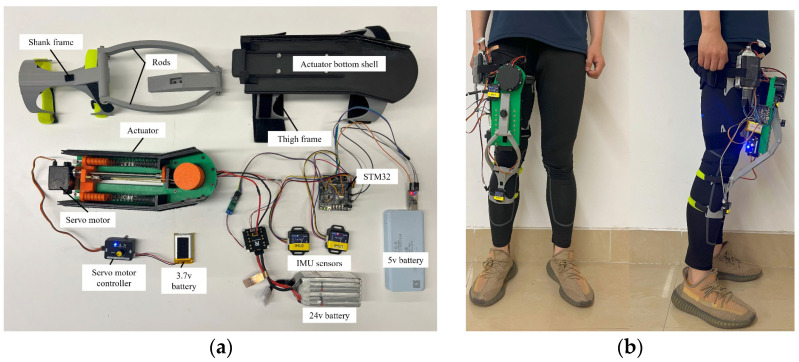
The exoskeleton prototype: (**a**) exoskeleton system components; (**b**) wearing diagram of the knee exoskeleton prototype.

In order to evaluate the overall working performance of the bionic knee exoskeleton, a wearable experiment is performed. All components of the exoskeleton system are processed and set up as shown in Figure 14a. Most of the parts of the exoskeleton are made by 3D printing. The motor uses a powerful robot joint motor as the driving source and is bolted to the upper housing of the actuator. The actuator bottom shell is fastened to the thigh clamp. Multiple batteries are used for the power supply, and DJI’s RoboMaster board is used to manage the power lines and CAN lines.

As shown in Figure 14b, the front and side views of the experimenter wearing the knee exoskeleton are presented. Two IMU sensors are placed on the shank frame and the upper housing of the actuator, respectively. The battery and control boards are placed on the higher side of the thigh frame to make the overall center of gravity of the device closer to the center of gravity of the human body, thereby improving the comfort of wearing the exoskeleton. During the experiment, three participants conducted the exoskeleton wearable experiment: the first participant was 165 cm tall and weighed 68 kg; the second was 185 cm tall and weighed 82 kg; and the third was 159 cm tall and weighed 50 kg. The experiment was conducted with a variety of movement patterns, including walking, running, climbing uphill, and taking stairs. The entire experiment was documented through a camera.

**Figure 15:**
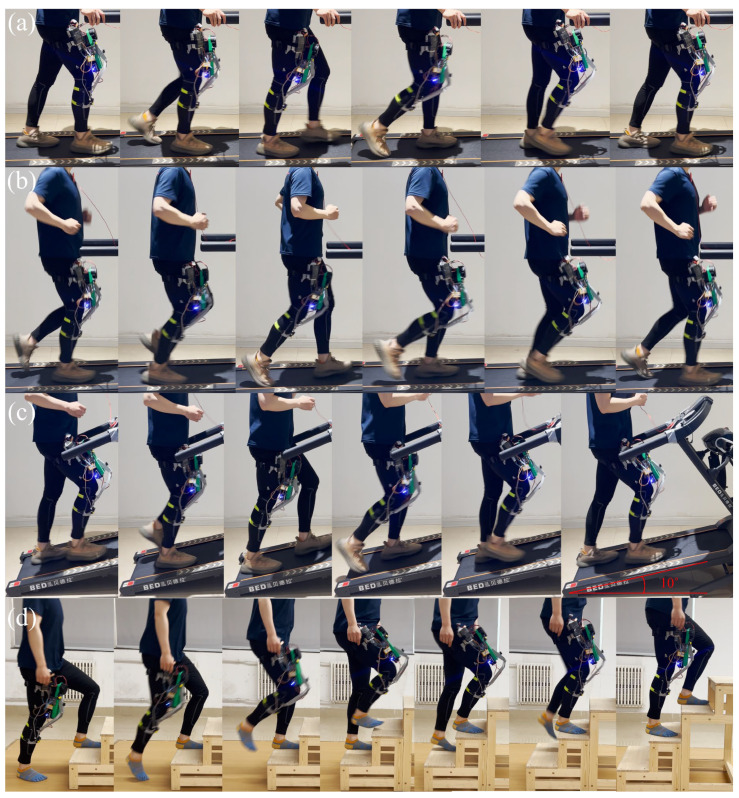
The experimental process: (**a**) walking experiment; (**b**) running experiment; (**c**) uphill walking experiment; (**d**) climbing stairs experiment.

As shown in Figure 15, the exoskeleton wearable experiment mainly tested four modes: walking, running, climbing uphill, and climbing stairs. For different modes, the clutch control drive system outputs power with different stiffness. The joint kinematics are presented through frame-by-frame extraction from the recorded video, and the observed motion trajectories are used to visually verify the synchronization and responsiveness of the exoskeleton joint motion with the user’s motion.

In the experiment, the walking speeds were set at 1 m/s and 1.5 m/s; the running speeds at 2.5 m/s and 3.5 m/s; the climbing walking speed at 1 m/s; the treadmill slope at 10 degrees; and the step height in the stair climbing experiment at 16 cm. To meet the biomechanical requirements of the human knee joint, as well as the body shape and strength of the exoskeleton wearer, and considering the mechanical properties of the aramid fiber ropes, the actuator uses four aramid fiber ropes with radii of 0.5 mm, 0.5 mm, 1 mm, and 2 mm, respectively. Multi-scenario testing of the compliance and comfort of the exoskeleton was carried out to select the appropriate actuator rope combination for each mode. At 1 m/s and 1.5 m/s in walking mode, we tested the effect of clutch intervention on a single 0.5 mm fiber rope and a 0.5 mm/0.5 mm fiber rope set. At 2.5 m/s and 3.5 m/s in running mode, we tried clutch intervention on a 0.5 mm/0.5 mm fiber rope set and a 0.5 mm/0.5 mm/1 mm rope set. At 1 m/s in climbing mode, the rope sets were 0.5 mm/0.5 mm, 2 mm, and 1 mm/2 mm, respectively. In the stair climbing test, the rope set selection was the same as for climbing.

**Figure 16:**
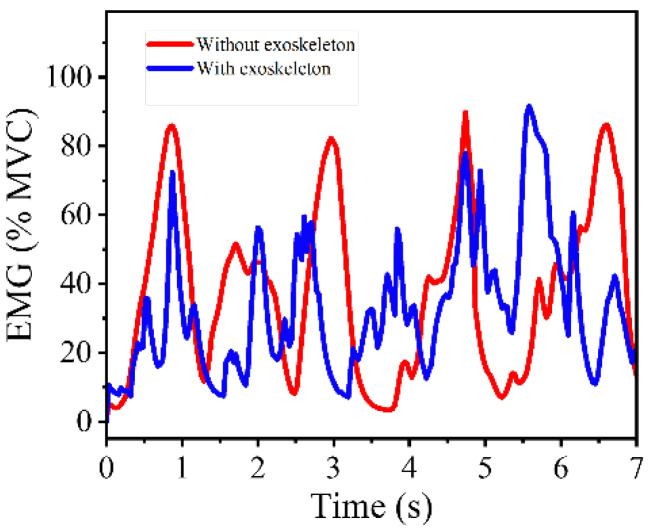
EMG experimental results.

During experimental testing, when walking at 1 m/s and 1.5 m/s, all participants reported that the drive mode with a single 0.5 mm aramid rope had the best compliance and wearing comfort. When running at 2.5 m/s and 3.5 m/s, participants of lower weight preferred the rope combination of 0.5 mm/0.5 mm, while participants of larger weight preferred the rope combination of 0.5 mm/0.5 mm/1 mm. In both the 1 m/s uphill climbing condition and upstairs climbing, all participants preferred the stiffer rope combination. According to the above performance, the following conclusions were drawn regarding the performance of different rope groups in different modes for different body types. When walking at low speed, a low-stiffness drive will bring better compliance and comfort. When running, the human body moves fast, and the joints need higher stiffness to maintain the stability of the body. The actuator should output power with higher stiffness. When climbing upstairs and walking uphill, the human body moves slowly but needs to overcome more gravitational potential energy to do work. High-stiffness power transmission reduces transmission delays and is conducive to body posture stability. In addition, we conducted an EMG experiment to compare muscle activation levels during walking with and without the knee exoskeleton. A lightweight, single-channel surface EMG sensor was placed on the rectus femoris to monitor muscle activity. As shown in Figure 16, the value of maximum voluntary contraction (MVC) over several gait cycles shows that muscle activation in the rectus femoris was reduced when the exoskeleton was worn, indicating that the system provides partial assistance to the knee joint and contributes to load sharing. The instability of EMG signals with exoskeleton may be caused by skin sweating or sensor compression. The latest Mechanomyography (MMG) technology is expected to improve this problem [35].

Overall, the stiffness and actuation characteristics of the knee exoskeleton prototype closely resemble the adaptive behavior of human muscles in modulating joint stiffness. The proposed actuator achieves adaptative driving stiffness across various motion modes and human body shapes, improves the compliance and comfort of the exoskeleton, and aligning with the bionic design goals of the knee exoskeleton prototype.

## 7. Conclusions

In this study, a bionic knee exoskeleton equipped with a variable stiffness actuator was developed, drawing inspiration from the adaptive stiffness modulation behavior of human muscles. By enabling dynamic switching between different rope bundle combinations, the system effectively modulates joint stiffness in response to varying gait requirements. The proposed mechanical structure, together with the mathematical modeling and experimental validation of the mechanical behavior of aramid fiber ropes, confirms the feasibility of the actuation system.

The implemented control framework successfully coordinates the variable stiffness actuation to adapt to different walking modes. Wearable experiments demonstrate that the exoskeleton improves user comfort and compliance by delivering appropriate stiffness levels tailored to specific gait phases. EMG results indicate that the exoskeleton provides a compensatory effect on the rectus femoris muscle. These findings suggest that bio-inspired stiffness modulation can significantly enhance the adaptability and wearability of lower limb exoskeletons, offering promising applications in rehabilitation and assistive locomotion.

## Figures and Tables

**Table 1:** Clutch activation sequence.

Serial Number	1	2	3	4	5	6	7
Activated ropes (mm)	0.5	0.5/0.5	0.5/0.5/1	0.5/0.5/1/2	0.5/1/2	1/2	2

**Table 2:** Parameter symbols and physical meanings.

Symbols	Physical Meanings
$D_{n}$	Prony series nth term coefficient
$\lambda_{n}$	The inverse of the nth term hysteresis time of the Prony series
$g_{0}$	Nonlinear instantaneous compliance coefficient of fiber rope stiffness
$g_{1}$	Nonlinear characteristic coefficient of fiber rope over-compliance
$g_{2}$	Parameters affecting load rate on deformation
$a_{\sigma }$	Acceleration factor, to characterize the coupling of stress and time in deformation
$D_{0}$	Instantaneous deformation flexibility
$D_{p}$	Plastic strain factor of the material (plastic strain rate)
*m*	Plastic strain factor of the material (material inherent property parameter)

**Table 3:** Aramid rope tension parameter.

**Rope diameter (mm)**	0.5	0.8	1.0	1.5	2.0	2.5
**Breaking tension (ton)**	0.08	0.13	0.16	0.24	0.31	0.4

**Table 4:** Experimental fitting coefficients.

Coefficients	Low Stress Level	High Stress Level
$g_{0}$	1	0.001294*x*^3^ − 0.09326*x*^2^ + 2.16*x* − 15.6
$g_{1}$	6.572 × 10^−6^*x*^−3^ − 0.001666*x*^−2^ + 0.13214*x* − 2.984	9.376 × 10^−6^*x*^3^ − 0.007052*x*^2^ + 0.1732*x* − 136.6
$g_{2}$	1	1.655 × 10^−6^*x*^3^ − 0.001318*x*^2^ + 0.3457*x* − 30.37
$a_{\sigma }$	1	2.895 × 10^−7^*x*^3^ − 0.0002084*x*^2^ + 0.04908*x* − 3.758
$D_{0}$	−0.0001236*x*^−3^ − 0.01641*x*^−2^ − 0.325*x* + 1.912	0.00751 × 10^−7^*x*^3^ − 0.6131*x*^2^ + 17.12*x* − 154
$D_{p}$	−1.307 × 10^−7^*x*^3^ − 4.596 × 10^−5^*x*^−2^ − 0.005338*x* + 0.2147	3.367 × 10^−7^*x*^3^ − 0.000261*x*^2^ + 0.0660261*x* − 5.41
*m*	1.174 × 10^−7^*x*^3^ − 0.0003098*x*^2^ − 0.02641*x* + 0.5669	−8.522 × 10^−7^*x*^3^ + 0.00064*x*^2^ − 0.1563*x* + 12.61

## Data Availability

The data are available upon request.
